# Socioecological Correlates of Park-based Physical Activity in Older Adults: A Comparison of Hong Kong and Leipzig Parks

**DOI:** 10.3390/ijerph16173048

**Published:** 2019-08-22

**Authors:** Ru Zhang, Yanping Duan, Walter Brehm, Petra Wagner

**Affiliations:** 1 Department of Sports Science & Physical Education, Faculty of Education, The Chinese University of Hong Kong, Hong Kong 999077, China; 2 Department of Sport and Physical Education, Faculty of Social Sciences, Hong Kong Baptist University, Hong Kong 999077, China; 3 Institute of Sport Science, Faculty of Humanities and Social Science, University of Bayreuth, 95447 Bayreuth, Germany; 4 Institute of Exercise and Public Health, Faculty of Sport Sciences, University of Leipzig, 04103 Leipzig, Germany

**Keywords:** park-based physical activity, older adults, socioecological model, urban parks, active living, perceived park environment

## Abstract

Aims: The present study aimed to examine and compare the socioecological correlates of park-based physical activity (PA) among Hong Kong and Leipzig older adults in terms of types of activity areas, perceived park environment, psychosocial factors, and the interactions between the perceived park environmental and psychosocial factors. Methods: Based on the SOPARC, systematic observations were conducted in six randomly selected urban parks in each city. A total of 317 Hong Kong (*M*_age_ = 69.96; *SD* = 6.81) and 311 Leipzig (*M*_age_ = 72.06; *SD* = 6.78) older adults from these six parks completed an interviewer-delivered survey measuring their perceived park environment, psychosocial variables, and park-based PA. Results: In both cities, the types of activity areas were positively associated with the number of older adults observed being active in parks. Psychosocial factors, especially self-efficacy and perceived barriers, had larger associations with park-based PA compared with the perceived park environment. The interaction between perceived park features and self-efficacy in the association with park-based PA was found in Leipzig, but not in Hong Kong. Conclusion: Findings of the current study contribute to the cross-cultural understanding of the socioecological correlates of park-based PA among older adults.

## 1. Introduction

Providing inexpensive and active resources in natural settings is a promising strategy to facilitate and increase physical activity (PA) [1]. In particular, urban parks have been identified as important spaces for people to be active in daily life [2]. An urban park is “an open area that is built by the local government for active and passive recreational uses” [3]. Although experimental research has proved PA in parks is related to several beneficial health outcomes in terms of cardiovascular disease, blood glucose, and mental health in comparison to the same amount of PA in non-green spaces [4,5], older adults do not use parks well for PA. Findings have revealed more than half of older adults never visit parks in daily life [6,7]. Additionally, less than 1/3 of older adults engage in PA in parks [8]. Therefore, it is important to understand the park-based PA of older adults and the possible determinants to increase health-related benefits.

The socioecological model is a widely used theoretical framework to explain PA and its potential determinants [9]. According to this model, park-based PA is influenced by factors from multiple levels, ranging from the park-built environment to individual factors such as the perceived park environmental and psychosocial variables [9]. Using the socioecological model to explain the determinants of park-based PA has highlighted some important park environment characteristics, especially the activity areas built-in parks such as walking trails and fitness corners [10]. The activity areas in parks indeed had stronger relationships with park-based PA compared to supporting amenities such as picnic tables [11]. Park-based PA could also be increased by improving the quality of activity areas such as playgrounds, walking tracks, and fitness corners [10,12]. To enhance park-based PA among older adults, studies are needed to assess how and which types of the activity areas built-in parks contribute to older adults’ active park use. With the exception of a few studies examining the relationships between self-reported park-based PA and the objectively measured presence of active facilities in American older adults [13], few studies examine the relationships between park-based PA and types of activity areas in older adults in other geospatial locations such as Asia and Europe [10]. 

According to the socioecological model, older adults’ perceptions of the park environment and psychosocial characteristics could influence their park-based PA [9]. Although the associations between some perceived park environments (e.g., park safety and attractiveness) and park-based PA were mixed [14], other perceived park environment factors (e.g., park features and access to parks) were found to be consistently associated with park-based PA [10]. The socioecological model also suggests correlates of PA interact across levels [9]. Therefore, it is important to examine how the perceived park environmental factors interact with psychosocial characteristic (e.g., self-efficacy and perceived benefits) when predicting park-based PA [15]. Although the interactions between physical environments and psychosocial factors have been supported for leisure-time walking [16,17], active transport [18], and total PA [18], the interactions between physical environments and psychosocial factors in the context of park-based PA have not been systematically examined until now, let alone the cross-cultural comparison of the interactions. 

Only a few studies have examined the generalizability of the socioecological model in a cross-cultural context, especially for park-based PA [19]. Most empirical research on park-based PA and the socioecological correlates was conducted in a single region with similar urban conditions [19]. The environmental data that were collected in a single context might have few variances and, in turn, underestimate the relationships [20,21]. Also, a large variety of sampling strategies and measurements used in different studies make it difficult to evaluate inter-region differences in individuals’ perception of park environment and their PA in parks [19]. From the cross-cultural perspective, systematic difference might exist for the activity areas between parks in different regions such as Hong Kong and Germany [22]. As such, examining data from different urban conditions (e.g., physical environments and population density) in different geospatial locations can increase the understanding about the sample-specific variations in the findings [19]. Comparable studies under different urban conditions can also narrow the methodological limitations by involving larger variance in the park-built environment to better evaluate the socioecological correlates of park-based PA [23]. Furthermore, people in collectivist cultures such as those from Asian countries tend to perceive themselves more interdependent and social cohesive, while people in individualist cultures such as those from Europe consider themselves to be self-contained and independent. Individuals from Asian and European regions (collectivism vs. individualism cultures) may have differences in perception of the park environment and social cognition [15]. Thus, it is needed to compare the cross-national differences in socioecological correlates of park-based PA such as the Asian and European regions [22]. 

### Aims of the Current Study

The aim of the current study was to examine and compare the socioecological correlates of park-based PA in older adults from Hong Kong and Leipzig. The cross-cultural data of the two cities were chosen and compared because Hong Kong and Leipzig have substantial differences in urban conditions and population density and show large variability in urban park environments [22]. Furthermore, these two cities have a similar proportion of older adults aged 65-plus, namely, 16.5% in Hong Kong [24] and 15% in Leipzig [25]. Per the tenets of the socioecological model and previous research [9,10,15], it was hypothesized that the socioecological model would be generalizable for explaining park-based PA among older adults in the two cities [9,19], specifically, park-based PA in parks among older adults in both cities would be associated with: H1) types of activity areas built-in parks [10]; H2) the perceived park environment characteristics including park safety, attractiveness, features, and proximity [6,14]; H3) psychosocial factors including self-efficacy, perceived benefits, perceived barriers, and enjoyment [9]; and H4) the interactions between the perceived park environment and the psychosocial factors [15]. Considering the differences in urban park environments and cultures across the two cities, we further hypothesized that there would be some sample-specific variations in the socioecological correlates of park-based PA between Hong Kong and Leipzig [19]. 

## 2. Methods

The current study involved both systematic observation and an interviewer-delivered survey in both Hong Kong and Leipzig parks from September 2014 to July 2015. A detailed description of the study design and sampling methods can be found elsewhere [22]. In brief, systematic observation was used to examine the association between types of activity areas and park-based PA (i.e., the number of older adults observed being active in parks). The interviewer-delivered survey was designed to ask older adults to report their perception of the park environment, social cognition, and park-based PA. 

### 2.1. Study Settings and Procedure

To balance between precision and time cost, we randomly selected 6 parks as study settings in Hong Kong and Leipzig, respectively. The criteria of park selection included (a) accessible parks that were not under construction [26]; (b) the parks represent varied geographic areas in the study cities [27]; and (c) the parks varied in size and activity areas [13]. All the potential areas that were likely to be used for PA were identified as activity areas [28]. We selected a total of 145 and 100 activity areas from the six Hong Kong and Leipzig parks, respectively. There were no significant differences in park size (*t* = −1.15, *p* = 0.28) or distribution of active areas (χ^2^ = 35.00, *p* = 0.24) across the parks in the two cities (see Table S1 in the online supplemental file).

Based on the manual of the System for Observation Play and Recreation in Communities (SOPARC) (28), the research group trained observers (i.e., student helpers) about how to conduct the objective and systematic observation. Observations by paired observers (i.e., student helpers) were required to reach a high level of inter-observer (IOA = 80%) and intraclass (r = 0.75) agreement after the training [28].

In each observation, systematic observations were conducted in the selected activity areas in the same order by the paired observers simultaneously and independently. The trained student helpers also conducted an interviewer-delivered survey for the older adults who were in the parks during the time of survey. To have a balanced sample, the older adults were randomly invited to participate in our study from the busiest and least-busy active spaces in the six Hong Kong and Leipzig parks. Gender balance was also considered during participant recruitment. Eligibility criteria included (a) aged 60 and over and (b) could walk without assistance. In total, we recruited 317 Hong Kong older adults (*M*_age_ = 69.96; *SD* = 6.81; ranging from 60 to 88 years old; 46.7% women) and 311 Leipzig older adults (*M*_age_ = 72.06; *SD* = 6.78; ranging from 60 to 92 years old; 58.5% women).

### 2.2. Measures

SOPARC is an observation tool measuring PA in natural spaces such as urban parks, and it has satisfactory validity and reliability [28]. Based on the manual of SOPARC, four key aspects of observation data were collected: (a) the number of sedentary park users, (b) PA types and intensity levels in activity areas, and (c) park users’ socio-demographics. To obtain a robust estimation of park-based PA among older adults, we conducted observations at four times daily (i.e., morning, noon, afternoon, and evening) on four different days (i.e., weekdays and weekends) in two seasons (i.e., fall and summer) during 2014–2015 [26,27]. A description of the SOPARC can be found in Table S2 in the online file of supplemental materials.

Self-reported questionnaires were used to investigate (a) older adults’ socio-demographics (i.e., gender, age, weight, height, education levels, and marital status), (b) psychosocial factors, (c) perceived park environments, and (d) park-based PA. Psychosocial and perceived park environment and park-based PA were measured using the assessment tools from previous research [17,29,30,31]. We measured five psychosocial factors, including self-efficacy (5 items), enjoyment (3 items), perceived benefits (3 items), perceived barriers (10 items), and social support (3 items) using a 5-point Likert scale ranging from “1” (strongly disagree) to “5” (strongly agree). For the perceived park environments, we measured park safety (4 items), attractiveness (4 items), and park features (4 items) using a 4-point Likert sale ranging from “1” (strongly disagree) to “4” (strongly agree). Perceived park distance (1 item) was measured using a 5-point Likert scale ranging from 1 (less than 5 min) to 5 (over 30 min). Furthermore, the participants were asked to report their frequency, duration, and intensity levels of PA in parks during a typical week. We calculated energy consumption of park-based PA (kcal/week) by multiplying metabolic equivalents (MET) values (kcal/min) and time (min/week). The questionnaires have been back-translated to Cantonese and German by 2 independent bilingual translators. Table S3 provides a description of the measures (e.g., reliability and sample item). 

### 2.3. Data Analysis

We evaluated the data using IBM SPSS Statistics 23 (Armonk, NY, USA; IBM Corp., 2015). Chi-square statistics were used to assess city differences in active older adults’ socio-demographics, activity levels, and use of activity areas. Negative binomial regression showed better model fit in the present study in comparison to Poisson regression. In addition, Poisson regression violated the assumption of equidispersion (the “Value/df” for the “Person Chi-Square was larger than 1). Therefore, negative binomial regression was used in the present study to test the relationships between types of activity areas and the number of older adults being active in parks. Park size, gender, day period, week period, and season were included as covariates in the analyses because these variables may affect park-based PA [28]. 

As previous studies suggested [32], the associations between park-based PA and each of the perceived park environment and psychosocial variables were analyzed first, and then the factors that showed a significant independent association with park-based PA were retained in the hierarchical multiple regression models. The socio-demographic variables that showed a significant correlation with park-based PA were included in the first block of the hierarchical regression model as covariates. In the second and third steps, the psychosocial and perceived park environment factors were included separately. Finally, the interaction terms between psychosocial and perceived park environment factors were included. To test the interaction terms, all the variables were mean-centered. For significant interaction terms, simple slope analyses were conducted to assess the association between park-based PA and psychosocial variables at low and high levels (+ 1 standard deviation) of the perceived park environment. 

### 2.4. Ethics Approval

Ethics approval and consent to participate: The current study involves human participants and the research protocol had been approved by the committee of Research Ethics and Safety (HASC) at Hong Kong Baptist University (No. FRG2/13-14/065). All participants provided written informed consent to participate.

## 3. Results

### 3.1. Descriptive Characteristics of the Samples

Table 1 shows descriptive characteristics of the Hong Kong and Leipzig older adults. In the systematic observation, a total of *N* = 3457 and *N* = 2665 older adults was observed in Hong Kong and Leipzig parks, respectively. We found differences in gender (χ^2^ (1) = 16.46, *p* < 0.001) and activity levels (χ^2^ (1) = 402.72, *p* < 0.001) between older adults in Hong Kong and Leipzig parks. Compared with older adults being observed in Hong Kong parks, a larger proportion of Leipzig older adults engaged in low- and moderate-PA in parks. Vigorous-intensity PA were more likely to be observed in Hong Kong older adults (20.6%) than those from Leipzig parks (3%). For the survey, compared to older adults from Hong Kong, older adults from Leipzig reported greater levels of self-efficacy, enjoyment, and social supports, and lower levels of perceived barriers. In addition, older adults from Leipzig perceived greater levels of park safety and attractiveness but reported larger distances from homes to parks. However, older adults from Hong Kong reported greater park-based PA than those from Leipzig, *t* = −2.16, *p* = 0.03.

### 3.2. Park-Based PA and Types of Activity Areas

As Table 2 shows, the association between park-based PA (i.e., the number of older adults observed being active in parks) and types of activity areas among Leipzig older adults was significant, Wald χ^2^ (4) = 928.95, *p* < 0.001, adjusting for park size, gender, time and week periods, and season. In comparison to the number of older adults observed in paths (i.e., the reference group), fewer Leipzig older adults were observed in playgrounds, sports fields, and fitness areas. Types of activity space were also positively associated with the number of active older adults in Hong Kong parks, Wald χ^2^ (6) = 538.18, *p* < 0.001. With paths as the reference group, playgrounds, sports fields, fitness areas, fastened areas (i.e., open spaces that has been paved), and skate parks were less likely to be used by the older adults observed in Hong Kong parks. H1 was supported in older adults from both Leipzig and Hong Kong parks.

### 3.3. Park-based PA and Perceived Park Environments

Table 3 describes the associations of park-based PA with each of the perceived park environment factors. Perceived park safety (*β* = 0.10, *p* = 0.11), attractiveness (*β* = 0.10, *p* = 0.09), park features (*β* = 0.01, *p* = 0.94), and park distance (*β* = −0.05, *p* = 0.38) did not have a significant relationship with park-based PA among older adults from Hong Kong parks, adjusting for age and gender. The findings indicated no perceived park environment factor was included in the hierarchical multiple regression model. Therefore, H2 cannot be supported using data of older adults from Hong Kong parks.

In terms of older adults from Leipzig parks, we found perceived park attractiveness (*β* = 0.14, *p* = 0.02), park features (*β* = 0.19, *p* = 0.001), and park distance (*β* = −0.14, *p* = 0.01) had a significant association with park-based PA, adjusting for marital status (see Table 3). Yet, the association between perceived park safety and park-based PA was not significant (*β* = 0.09, *p* = 0.12). Therefore, only perceived park attractiveness, park features, and park distance were added in the final hierarchical multiple regression model. As Table 4 shows, the three perceived park environment factors explained 3.1% of variance in park-based PA among Leipzig older adults (*F*(5, 271) = 6.96, *p* < 0.001). However, perceived park attractiveness (*β* = 0.02, *p* = 0.79), park features (*β* = 0.12, *p* = 0.07), and park distance (*β* = −0.10, *p* = 0.09) did not show significant associations with older adults’ park-based PA in Leipzig parks. As such, we found partial support for H2 in older adults from Leipzig parks.

### 3.4. Park-Based PA and Psychosocial Factors

Table 3 shows the associations between park-based PA and each of the psychosocial factors. For older adults from Hong Kong parks, park-based PA was significantly associated with self-efficacy (*β* = 0.31, *p* < 0.001), enjoyment (*β* = 0.31, *p* < 0.001), perceived benefits (*β* = 0.30, *p* < 0.001), and perceived barriers (*β* = −0.39, *p* < 0.001), although the association with social support was not significant (*β* = 0.01, *p* = 0.86). The influences of age and gender were adjusted. Perceived benefits, perceived barriers, self-efficacy, and enjoyment, were included in the hierarchical multiple regression model, with self-efficacy (*β* = 0.18, *p* < 0.01) and perceived barriers (*β* = −0.28, *p* < 0.001) showing significant associations with park-based PA (see Table 4). H3 was partially supported in older adults from Hong Kong parks.

Similar findings were also revealed in older adults from Leipzig parks. Self-efficacy (*β* = 0.21, *p* < 0.001), enjoyment (*β* = 0.22, *p* < 0.001), perceived benefits (*β* = 0.14, *p* = 0.01), and perceived barriers (*β* = −0.31, *p* < 0.001) showed significant associations with park-based PA (see Table 3). Self-efficacy (*β* = 0.14, *p* = 0.02) and perceived barriers (*β* = −0.25, *p* < 0.001) predicted the park-based PA in the multiple regression model (Table 4). These findings indicated H3 was also partially supported in older adults from Leipzig parks.

### 3.5. Interactions between Perceived Park Environment and Psychosocial Factors

**Figure 1:**
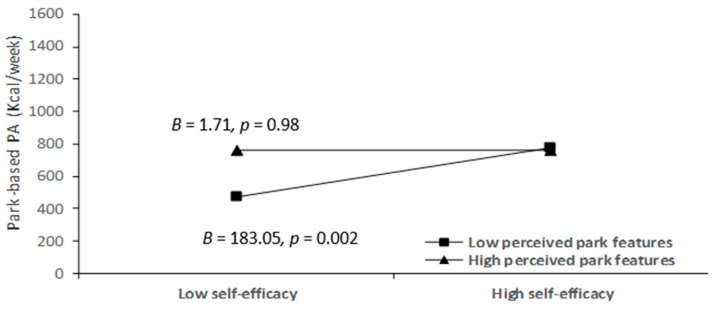
The interaction between perceived park features and self-efficacy in the association with park-based physical activity among Leipzig older adults.

As Table 4 shows, only one of the 12 interaction terms (i.e., perceived park features * self-efficacy) was significantly associated with park-based PA among older adults in Leipzig, *β* = −0.15, *p* = 0.03. As Figure 1 shows, the results of simple slopes analyses indicated self-efficacy was positively associated with park-based PA at the low level (+ 1 standard deviation) of perceived park features (*β* = 0.28, *t* (259) = 3.14, 95% CI (68.36, 297.73), *p* = 0.002), while the association was not significant at the high level (−1 standard deviation) of park features, *β* = 0.003, *t* (259) = 0.03, 95% CI (−112.03, 115.46), *p* = 0.98. H4 was partially supported in older adults from Leipzig parks. However, it was not supported in older adults from Hong Kong parks because neither perceived park environments nor interaction terms can predict park-based PA.

## 4. Discussion

The present study aimed to examine and compare the socioecological correlates of park-based PA among older adults from both Hong Kong and Leipzig parks in terms of types of activity areas, perceived park environment, psychosocial factors, and the interactions between the perceived park environments and psychosocial factors. Significant associations between types of activity areas and park-based PA (i.e., number of older adults observed being active in parks) were found in both cities. Findings provided partial support for the associations of the perceived park environments and psychosocial variables and their interactions with park-based PA among older adults from both the Hong Kong and Leipzig parks. 

The results of our study identified the importance of activity areas, especially the paths facilitating PA for older adults in parks. We found types of activity areas were positively associated with the number of older adults observed in Hong Kong and Leipzig parks. These findings align with previous studies which found that providing activity facilities in parks can promote individuals’ active park use [10,33,34]. We also found the older adults in both Hong Kong and Leipzig parks consistently preferred to use paths compared with the other types of activity areas. This finding is in line with previous findings in which using paths for walking was the most common PA type for older adults [35]. Our findings indicate building elder-friendly activity areas in parks, especially paths, might be a better strategy for improving older adults’ park-based PA. 

Although park attractiveness, park distance, and park features were significantly associated with park-based PA among older adults in Leipzig, none of the perceived park environment correlates were found in Hong Kong older adults. The substantial variation in perceptions of physical environment has been revealed in previous research [2]. The different results may reflect variations in the cultural, social, and park-built environments of the two cities [21]. Park environments in Leipzig probably play a more important role for facilitating older adults’ PA than in Hong Kong, where parks are considered to be equally accessible and elder-friendly and the low variability in the park environment may hamper the environment-PA relationship [20,36]. An additional explanation for the non-significant environment-PA relationship is that a larger amount of variance in park-based PA was explained by psychosocial factors compared with the perceived park environment factors. This might indicate the perceived environmental factors have little effect on PA beyond individuals’ social cognition [16]. Future research might consider investigating a larger number of parks as well as combining objectively measured and perceived physical environments to examine park-based PA across different geospatial locations.

Regarding the psychosocial correlates of park-based PA, self-efficacy and perceived barriers were consistently associated with park-based PA among older adults in both cities. Significant positive association between self-efficacy and park-based PA aligns with previous studies demonstrating the improvement of self-efficacy on increasing older adults’ PA in leisure time [18,37]. It might indicate older adults who are commonly active in parks are those with high self-efficacy for PA. The negative relationship of perceived barriers with park-based PA is consistent with the qualitative findings that some barriers such as health problems and time constraints may hamper older adults’ park use for PA [35]. Future studies might consider exploring what kinds of barriers inhibit older adults from participating in park-based PA and developing the behavior change interventions targeting the increase of older adults’ self-efficacy and the decrease of their perceived barriers [38].

Findings on older adults from Leipzig parks demonstrated perceived park features moderated the relationship between self-efficacy and park-based PA. We found self-efficacy had greater effects on park-based PA in the older adults with lower perception of park features, whereas, for those with greater perceived park features, the effects of self-efficacy on park-based PA did not reach significance. Similar patterns for the interaction between self-efficacy and park features have been found in recreational walking [17] and moderate-to-vigorous PA [39]. A possible understanding of these results is that older adults with great satisfaction with park features prefer to use parks for PA, regardless of the levels of their self-efficacy [15]. It is, therefore, suggested older adults with high self-efficacy may be easily cued by elder-friendly park environments to act according to their motives and, in turn, contribute to the increase of park-based PA. Future park-based PA promotion intervention for older adults might consider how to increase their self-efficacy levels based on social cognitive theory [38]. 

Findings of the current study indicated that there were some variations in park-based PA and the socioecological correlates between Hong Kong and Leipzig [19]. The current study observed a larger proportion of Leipzig older adults engaging in low-and moderate-intensity PA than those from Hong Kong parks, while the proportion of vigorous-intensity PA was larger in Hong Kong parks than Leipzig parks. This indicates that older adults in Leipzig parks did not perform PA as highly as did older adults in Hong Kong parks. The variation in park-based PA could be explained by the differences in living contexts between the two cities. Compared with Leipzig, Hong Kong has extra higher population density and walkability, making urban parks in Hong Kong more accessible for older adults to use. An additional understanding for this variation is the ethnic and cultural differences between Hong Kong and Leipzig. Collectivist contexts like those from Hong Kong tend to engage in group activities such as Tai chi and dancing in parks and have more social considerations when visiting parks [22]. Compared with Hong Kong older adults, older adults in individualist cultures such as German are more likely to participate in individual activities and make decisions from personal considerations like self-efficacy, enjoyment, and attitudes [15]. 

Furthermore, substantial differences were revealed in the perceived park environment correlates of park-based PA between the two cities. The differences in perceived park environmental correlates of park-based PA across the two cities support our hypothesis the geographic differences in the two cities may influence some sample-related variations in the socioecological correlates. These findings align with previous studies in which perception of physical environments varied by regions [40], which can be explained by the differences in population density and the park-built environment across diverse regions [22]. Considering the small sample size of the current study may underestimate park-based PA and the socioecological correlates, future research is needed to have a large sampling design to examine the cross-cultural differences.

### Study Limitations and Strengths

Limitations of this study need to be acknowledged. First, the cross-sectional design makes it difficult to infer any causal relationship between park-based PA and the socioecological correlates. Further research is needed to conduct quasi-experimental or longitudinal investigations, by which the causal inferences could be reached [8]. Second, when counting the numbers of (active) park users, the inter-observer consistency may lead to misclassification bias using SOPARC. Therefore, in line with the manual of SOPARC [28], all the observers were well trained and reached satisfactory inter-observer agreement. Third, the impacts of selection bias on older adults’ park-based PA cannot be excluded given the cross-sectional nature of the current study because “selection bias” might occur when people decide to live in the places with active resources [41]. The “selection bias” may bring an overestimation of park environments because older adults consider urban parks as recreational resources for active lifestyles. Longitudinal and experimental design should be considered in the future to eliminate the influences of bias in selection [8]. Finally, this study recruited s small sample from limited urban parks in the Hong Kong and Leipzig. Future research should have a larger sampling design for a robust estimation.

The study also has several strengths. First, we used a multi-method research design including systematic observation and interviewer-delivered surveys to estimate the multiple influences on park-based PA. Second, the association between types of activity areas and number of active older adults was objectively examined using SOPARC, which offers its ability to objectively evaluate PA in park in terms of PA types, intensity levels, and temporal habits, instead of using self-reported measures that may induce response bias [7,28]. Third, we assessed older adults’ perception of the park environment in relation to their park-based PA. Compared with objective data about the park environment, findings of the perceived park environment show greater advantage for us to understand park-based PA because older adults could report a value judgment regarding the park environment and, in turn, their perceptions may influence their decisions and behaviors [6].

## 5. Conclusions

This study is the first cross-regional study examining and comparing socioecological correlates of park-based PA among older adults from Hong Kong and Leipzig parks. The findings supported the associations of activity area types, especially paths, with older adults’ park-based PA. Psychosocial factors of self-efficacy and perceived barriers compared to the perceived park environments showed larger influence on park-based PA among older adults from Leipzig parks than older adults from Hong Kong parks. The interaction between perceived park feature and self-efficacy when explaining park-based PA was found in older adults from Leipzig parks but not in older adults from Hong Kong parks. Practically speaking, our findings can inform the natural experimental studies using parks to promote active aging on increasing older adults’ park-based PA. Findings of the current study contribute to the cross-cultural understanding of the socioecological correlates of park-based PA among older adults.

## Figures and Tables

**Table 1:** Descriptive statistics for the observed and survey sample characteristics by city.

	Hong Kong	Leipzig	*χ*^2^/*t*	*p*
**SOPARC observation**				
Total observed older adults, *N*	3457	2665		
**Gender, *n* (%)**				
Males	2006 (58.0%)	1408 (52.8%)	16.46	<0.001
Females	1451 (42.0%)	1257 (47.2%)		
**Ethnicity, *n* (%)**				
White	18 (0.5%)	2624 (98.5%)	–	–
Asian	3439 (99.5%)	29 (1.1%)		
Other	0 (0)	12 (0.5%)		
**Activity levels, *n* (%)**				
Mild	1693 (49.0%)	1296 (48.6%)	484.27	<0.001
Moderate	1052 (30.4%)	1288 (48.3%)		
Vigorous	712 (20.6%)	81 (3.0%)		
**Use of activity areas, *n* (%)**				
Playgrounds	320 (9.3%)	50 (1.9%)	–	–
Lawn spaces	39 (1.1%)	48 (1.8%)		
Fitness areas	725 (21.0%)	12 (0.5%)		
Sports fields	547 (15.8%)	2 (0.1%)		
Skateparks	2 (0.1%)	–		
Fastened spaces	138 (4.0%)	–		
Paths	1686 (48.8%)	2553 (95.8%)		
**Interviewer-delivered survey**				
Total participants, *N*	317	311		
**Psychosocial factors, *M* (SD)**				
Self-efficacy ^a^	3.05 (0.95)	3.43 (0.83)	5.22	<0.001
Enjoyment ^b^	3.93 (0.80)	4.70 (0.60)	13.45	<0.001
Perceived benefits ^b^	3.73 (0.64)	3.83 (0.70)	1.84	0.07
Perceived barriers ^b^	2.27 (0.78)	1.43 (0.38)	−16.82	<0.001
Social support ^b^	2.82 (0.96)	3.17 (1.29)	3.83	<0.001
**Perceived park environment, *M* (SD)**				
Park safety ^c^	3.34 (0.59)	3.48 (0.55)	3.11	0.002
Attractiveness ^c^	3.07 (0.53)	3.44 (0.41)	9.99	<0.001
Park features ^c^	3.22 (0.54)	3.24 (0.58)	0.56	0.58
Park distance ^d^	2.02 (0.95)	2.27 (1.11)	3.11	0.002
**Self-reported park-based PA** (Kcal/week), *M* (SD)	859.11 (790.17)	737.22 (571.22)	−2.16	0.03

Note. *M* = Mean; *SD* = Standard deviation; PA = Physical activity. Row percentages may not be 100% due to rounding. – Not relevant. ^a^: from 1 (*I am sure I cannot*) to 5 (*I am sure I can*); ^b^: from 1 (*strongly disagree*) to 5 (*strongly agree*); ^c^: from 1 (*strongly disagree*) to 4 (*strongly agree*); ^d^: from 1 (*less than 5 min*) to 5 (*more than 30 min*).

**Table 2:** Results of negative binominal regression analyses assessing the associations between number of older adults observed being active in parks and the types of activity areas.

Factors	Hong Kong (*n* = 1232)			Leipzig (*n* = 704)		
	*OR*	(95% CI)	*p*	*OR*	(95% CI)	*p*
Covariates						
**Park size**						
Small	1.18	(1.09, 1.28)	<0.001	0.85	(0.76, 0.95)	0.004
Big	(1.00)	–	–	(1.00)	–	–
**Gender**						
Male	0.98	(0.91, 1.07)	0.69	1.07	(0.96, 1.21)	0.23
Female	(1.00)	–	–	(1.00)	–	–
**Time periods**						
Morning	1.21	(1.10, 1.34)	<0.001	0.87	(0.74, 1.03)	0.11
Noon	1.11	(0.99, 1.23)	0.07	0.92	(0.79, 1.07)	0.29
Afternoon	1.24	(1.11, 1.38)	<0.001	1.25	(1.07, 1.47)	0.01
Evening	(1.00)	–	–	(1.00)	–	–
**Week periods**						
Weekday	1.02	(0.95, 1.10)	0.60	0.79	(0.71, 0.88)	<0.001
Weekend	(1.00)	–	–	(1.00)	–	–
**Seasons**						
Fall	1.04	(0.96, 1.12)	0.37	0.95	(0.84, 1.06)	0.34
Spring	(1.00)	–	–	(1.00)	–	–
Main effects						
**Activity areas**						
Playgrounds	0.35	(0.31, 0.40)	<0.001	0.26	(0.24, 0.29)	<0.001
Lawn spaces	0.87	(0.54, 1.42)	0.58	0.74	(0.36, 1.55)	0.43
Fitness areas	0.34	(0.31, 0.38)	<0.001	0.33	(0.23, 0.46)	<0.001
Sports fields	0.45	(0.40, 0.51)	<0.001	0.25	(0.22, 0.29)	<0.001
Skateparks	0.40	(0.35, 0.45)	<0.001	–	–	–
Fastened spaces	0.56	(0.45, 0.71)	<0.001	–	–	–
Path	(1.00)	–	–	(1.00)	–	–

*Note*. *OR* = Odds ratio; 95% CI = 95% confidence interval. – Not relevant.

**Table 3:** Results of the associations between park-based physical activity and each of the perceived park environmental and psychosocial factors in older adults from Hong Kong and Leipzig.

Predictors	*B(SE)*	*β*	95% CI
*Hong Kong (n = 270–279) ^a^*			
**Perceived park environment**			
Park safety	124.68 (76.68)	0.10	(−26.29, 275.64)
Attractiveness	145.34 (86.23)	0.10	(−24.43, 315.10)
Park features	6.69 (86.02)	0.01	(−162.67, 176.04)
Park distance	−43.10 (49.08)	−0.05	(−139.72, 53.52)
**Psychosocial factors**			
Self-efficacy	251.18 (45.68)	0.31 ***	(161.25, 341.11)
Enjoyment	304.61 (55.71)	0.31 ***	(194.92, 414.29)
Perceived benefits	365.00 (70.09)	0.30 ***	(227.00, 502.99)
Perceived barriers	−400.02 (57.08)	−0.39 ***	(−512.40, −287.63)
Social support	8.80 (48.94)	0.01	(−87.55, 105.15)
*Leipzig (n = 296–304) ^b^*			
**Perceived park environment**			
Park safety	96.00 (60.76)	0.09	(−23.58, 215.57)
Attractiveness	195.81 (80.76)	0.14 *	(36.88, 354.75)
Park features	184.89 (54.42)	0.19 **	(77.79, 291.98)
Park distance	−73.36 (29.15)	−0.14 *	(−130.73, −15.99)
**Psychosocial factors**			
Self-efficacy	143.69 (39.02)	0.21 ***	(66.91, 220.47)
Enjoyment	208.22 (53.72)	0.22 ***	(102.50, 313.93)
Perceived benefits	116.13 (46.96)	0.14 *	(23.72, 208.55)
Perceived barriers	−443.56 (82.46)	−0.31 ***	(−605.85, −281.27)
Social support	30.92 (26.50)	0.07	(−21.23, 83.07)

*Note*. *B* = Unstandardized coefficients; *SE* = Standard error; 95% CI = 95% confidence interval. ^a^: Control variables in Hong Kong included age and gender; ^b^: Control variables in Leipzig included marital status; * *p* < 0.05, ** *p* < 0.01, *** *p* < 0.001, 2 tailed.

**Table 4:** Results of the hierarchical multiple regression analysis predicting the perceived park environment, psychosocial factors and their interactions in the associations with park-based PA among older adults from Hong Kong and Leipzig.

Predictors	Hong Kong (*n* = 261)			Leipzig (*n* = 280)		
	*B(SE)*	*β*	95% CI	*B(SE)*	*β*	95% CI
Block1: Control variables						
Gender	−185.02 (86.02)	−0.12 *	(−354.42, −15.63)	–	–	–
Age	7.27 (6.39)	0.06	(−5.31, 19.85)	–	–	–
Marital status	–	–	–	−23.54 (36.02)	−0.04	(−94.47, 47.40)
Block2: Psychosocial factors						
Self-efficacy	140.51 (50.60)	0.18 **	(40.87, 240.15)	92.38 (39.72)	0.14 *	(14.16, 170.60)
Enjoyment	54.26 (76.78)	0.06	(−96.95, 205.47)	28.19 (70.81)	0.03	(−111.24, 167.62)
Perceived benefits	108.99 (88.42)	0.09	(−65.13, 283.11)	40.14 (49.27)	0.05	(−56.87, 137.16)
Perceived barriers	−283.92 (67.68)	−0.28 ***	(−417.21, −150.63)	−342.91 (94.04)	−0.25 ***	(−528.09, -157.72)
Block3: Perceived park environment						
Park features	–	–	–	116.90 (63.48)	0.12	(−8.10, 241.91)
Park distance	–	–	–	−49.27 (28.98)	−0.10	(−106.32, 7.78)
Attractiveness	–	–	–	24.19 (91.11)	0.02	(−155.23, 203.61)
Block 4: Interactions			–	–		
Attractiveness × self-efficacy	–	–	–	−13.98 (102.93)	−0.01	(−216.66, 188.70)
Attractiveness × enjoyment	–	–	–	92.50 (168.29)	0.05	(−238.88, 423.89)
Attractiveness × perceived benefits	–	–	–	−177.66 (138.32)	−0.12	(−450.04, 94.72)
Attractiveness × perceived barriers	–	–	–	−8.28 (239.49)	−0.003	(−479.87, 463.31)
Park distance × self-efficacy	–	–	–	−15.72 (33.30)	−0.03	(−81.30, 49.87)
Park distance × enjoyment	–	–	–	31.49 (60.27)	0.04	(−87.19, 150.17)
Park distance × perceived benefits	–	–	–	−74.53 (48.60)	−0.11	(−170.24, 21.18)
Park distance × perceived barriers	–	–	–	29.37 (83.47)	0.02	(−135.00, 193.75)
Park features × self-efficacy	–	–	–	−157.63 (73.48)	−0.15 *	(−302.33, -12.94)
Park features × enjoyment	–	–	–	−4.26 (160.35)	−0.003	(−320.01, 311.50)
Park features × perceived benefits	–	–	–	67.04 (103.03)	0.05	(−135.85, 269.92)
Park features × perceived barriers	–	–	–	−177.80 (186.25)	−0.09	(−544.56, 188.97)

*Note*. *B* = Unstandardized coefficients; *SE* = Standard error; *β* = Standardized coefficient; 95% CI = 95% confidence intervals. – Not relevant. * *p* < 0.05, ** *p* < 0.01, *** *p* < 0.001, 2 tailed.

## Supplementary Materials

The following are available online at https://www.mdpi.com/1660-4601/16/17/3048/s1. Table S1: Urban park environment in Hong Kong and Leipzig; Table S2: Constructs and description used in the observation analyses; Table S3: Measures used to assess the perceived park environment, psychosocial factors and park-based physical activity.
