# Supplementary material for: Socioecological Correlates of Park-based Physical Activity in Older Adults: A Comparison of Hong Kong and Leipzig Parks
Source: Int J Environ Res Public Health. 2019 Aug 22;16(17):3048. doi: 10.3390/ijerph16173048 (PMC6747084; doi:10.3390/ijerph16173048)
Supplement: Supplementary file 1 [file ijerph-16-03048-s001.pdf]

Supplemental File

Table S1: Urban park environment in Hong Kong and Leipzig

Table S2: Constructs and description used in the observation analyses

Table S3: Measures used to assess the perceived park environment, psychosocial factors and park-based physical activity

Table S1. Urban park environment in Hong Kong and Leipzig.

**Park environment**	**Hong Kong**	**Leipzig**	t/χ^2
Park size, *M* (*SD*)	11.17 (5.91)	19.68 (17.21)	−1.15
Activity areas, *n* (%)			
Playgrounds	22 (15.2%)	28(28.0%)	
Lawn spaces	3 (2.1%)	32(32.0%)	
Fitness areas	30 (20.7%)	2 (2.0%)	
Sports fields	73 (50.3%)	24(24.0%)	35.00
Skateparks	2 (1.4%)	2 (2.0%)	
Fastened spaces	7 (4.8%)	0 (0.0%)	
Paths	8 (5.52%)	12(12.0%)	

Table S2. Constructs and description used in the observation analyses.

Variables	Description	Variable types
**Types of activity areas**		Dummy variable
Sedentary park users	Number of observed park users who are sedentary during observation	Count variable
**Park-based PA**		
Types	Specific types of physical activity that are observed in activity spaces in parks	Dummy variable
Intensity levels	Number of observed park users being active in the low, moderate or vigorous intensity levels during observation (Ainsworth et al., 2000).	Count variable
**Socio-demographics variables**		
Age	Number of older adults doing PA during observation	Count variable
gender	Number of male and female older adults dong PA during observation	Count variable
Social situation	Number of observed park users being active in individual or in group	Count variable
Ethnicity	Number of White, Asian and others doing PA during observation	Count variable
**Temporal factors**		
Time periods	Time periods of observation, including 8:30am, 11:00am, 15:00am and 17:30pm	Dummy variable
Week periods	Days of observation, including weekdays and weekends	Dummy variable
Seasons	Seasons of observation, including fall in 2014 and spring in 2015	Dummy variable

Table S3. Measures used to assess the perceived park environment, psychosocial factors and park-based physical activity.

Construct	Item example and description	Response scale	Internal reliability (Cronbach's α)
	**Psychosocial factors**		
Self-efficacy (5 items)	I am confident that I can participate physical activity in parks even when I am tired.	1–5 [a]	Hong Kong: 0.88; Leipzig: 0.72
Enjoyment (3 items)	I enjoy doing physical activities in parks.	1–5 [b]	Hong Kong: 0.92; Leipzig: 0.87
Perceived benefits (10 items)	If I participate physical activity in parks, then I will feel less depressed and/ or bored.	1–5 [b]	Hong Kong: 0.93; Leipzig: 0.84
Perceived barriers (15 items)	Self-conscious about my looks when I exercise.	1–5 [b]	Hong Kong: 0.95; Leipzig: 0.82
Social support (3 items)	My family do physical activity in urban parks with me.	1–5 [b]	Hong Kong: 0.86; Leipzig: 0.89
	**Perceived park environmental factors**		
Park safety (4 items)	It is safe to engage in physical activity in parks.	1–4 [c]	Hong Kong: 0.82; Leipzig: 0.71
Attractiveness (4 items)	This park is a pleasant environment for walking, running and other physical activities.	1–4 [c]	Hong Kong: 0.70; Leipzig: 0.46
Park features (4 items)	The facilities for physical activity in this park are in a good condition.	1–4 [c]	Hong Kong: 0.85; Leipzig: 0.73
Park distance (1 item)	"How long would it take to get from your home to this park?", Participants were asked to answer by "<5mis", "6-10mins", "11-20mins", "21-30mins" or ">30mins"	1–5 [d]	–
	**Self-reported park-based physical activity**		
Frequency	Participants were asked "What is the normal frequency of physical activities you usually are engaged in parks?" 6 options include "Once per month or less", "Two to three times per month", "Up to 1 hour per week", "More than 1 till 2 hours per week", "More than 2 till 4	–	–

Construct	Item example and description	Response scale	Internal reliability (Cronbach's α)
	hours per week" and "More than 4 hours per week". Frequency was corresponded to accumulated time.		
Intensity level	Participants were asked "What is the normal intensity of physical activities you usually are engaged in parks?" 3 options include "mild (no sweating and no shortness of breath)", "moderate (some sweating and /or some shortness of breath)" and "vigorous (heavy sweating and/or heavy shortness of breath). Intensity levels were transferred to relevant MET values (Ainsworth et al., 2000).	–	–
Energy consumption of park-based PA	Energy consumption of park-based PA (kcal/week) was calculated by multiplying time (min/week) and Met values (kcal/min)	–	–

Note. – Not relevant. [a]: from 1 (*I am sure I cannot*) to 5 (*I am sure I can*); [b]: from 1 (*strongly disagree*) to 5 = (*strongly agree*); [c]: from 1 = (*strongly disagree*) to 4 = (*strongly agree*); [d]: from 1 = (*less than 5 minutes*) to 5 (*more than 30 minutes*).
